# Clinical Neuropathology Views – 2/2016: Digital networking in European neuropathology: An initiative to facilitate truly interactive consultations 

**DOI:** 10.5414/NP300899

**Published:** 2016-02-02

**Authors:** Miguel A. Idoate, Marcial García-Rojo

**Affiliations:** 1 Department of Pathology, University of Navarre, Pamplona and; 2 Department of Pathology, Hospital of Jerez, Jerez de la Frontera, Cádiz, Spain

**Keywords:** digital networking, neuropathology, interactive consultations, European networking, telemedicine, telepathology

## Abstract

Digital technology is progressively changing our vision of the practice of neuropathology. There are a number of facts that support the introduction of digital neuropathology. With the development of whole-slide imaging (WSI) systems the difficulties involved in implementing a neuropathology network have been solved. A relevant difficulty has been image standardization, but an open digital image communication protocol defined by the Digital Imaging and Communications in Medicine (DICOM) standard is already a reality. The neuropathology network should be established in Europe because it is the expected geographic context for relationships among European neuropathologists. There are several limitations in the implementation of a digital neuropathology consultancy network such as financial support, operational costs, legal issues, and technical assistance of clients. All of these items have been considered and should be solved before implementing the proposal. Finally, the authors conclude that a European digital neuropathology network should be created for patients’ benefit.

## Digital neuropathology: Is it really needed? 

Digital technology is progressively changing our vision of the practice of neuropathology. The usefulness of digital technology in medicine has already been well demonstrated through consensus reviews, quality assurance, tissue microarrays, education, and proficiency testing. It is foreseeable that neuropathology departments will progressively incorporate this technology into their routine practice. 

The greatest potential of digital neuropathology will be achieved when neuropathologists decide to make substantial changes in the way they work by introducing digital technology. Certainly, implementation of quantitative image analysis will permit professionals to obtain more comprehensive and precise neuropathological reports. 

Digital neuropathology already plays an important role in teaching and research, and its uses are growing exponentially [1]. Digital neuropathology is already commonly used in the research field and greatly facilitates interactions among researchers, whereas in teaching it provides an excellent tool for medical students to integrate neuropathology with clinical medicine [2]. The interactive digital approach to studying cases appears to be particularly valuable in neuropathology teaching [3]. However, the implementation of digital neuropathology in clinical practice is just starting, and a great deal of skepticism seems to preclude its introduction in routine diagnostic work. For those who have experience in large-scale pathological diagnoses, the transition from conventional light microscopy to a digital-based workflow for imaging potentially offers improved efficiency and related operational cost savings [4]. 

In the coming years, the application of image analysis in neuropathology is likely to continue to increase, making this a critical area of development for our specialty. 

## Reasons for a network in neuropathology 

Frequently, hospitals in a variety of European countries have neither specialized personnel nor adequate technology to confront clinical neuropathology challenges. Additionally, the problem may be compounded by a low neuropathology case workload and the reluctance of general pathologists to get involved. 

Other facts that support the introduction of digital neuropathology are the following: 

Firstly, digital neuropathology permits professionals to properly address the quantitative aspects of diagnosis [5]. 

Secondly, digital neuropathology makes tissue bank specimens widely accessible to neuropathologists. This is particularly relevant in the field of neurodegeneration. Now, digital slides offer a better selection and classification of tissue, since complete digital slides offer more information than static pictures. 

Thirdly, digital neuropathology increases the ability to engage and collaborate with experts. Neuropathologists often feel the need to discuss difficult cases with their colleagues, but whereas the traditional system of sending slides is a very slow and laborious procedure, consultation is greatly enhanced by digital neuropathology. 

Fourthly, digital neuropathology permits professionals to share limited biopsies that can now be submitted as a single slide for scanning and can be presented to all participants in a networking approach. Currently, this technological revolution allows a number of cases to be available for viewing and interpretation by any neuropathologist in the network, regardless of location. 

## Is a digital neuropathology network a good solution? 

With the development of whole-slide imaging (WSI) systems, which allow for the evaluation and interpretation of digital images of entire histologic sections [7], the difficulties in implementing a neuropathology network have been solved. This technology is much more accessible and popular now than ever [8]. Nowadays, the WSI slide is easy to handle. 

One of the more relevant difficulties has been image standardization. Relevant efforts have been made to solve this problem. Currently, the diagnostic information contained in a digital slide obtained from a given scanner may not be translatable to another scanner. Furthermore, image analysis algorithms may need to be modified substantially to work on images created by different scanner types. To avoid this problem, an open digital image communication protocol defined by the Digital Imaging and Communications in Medicine (DICOM) standard is already a reality. Therefore, the best solution would be to adapt scanners to use this standard for the sake of international consultancy. 

DICOM has been recommended by the international guidelines of Telepathology and Digital Pathology (Digital Pathology Association, Canadian Association of Pathologists, and Spanish Society of Pathologists). In 2015, DICOM compliance was already announced by at least two scanning companies. It is true that this initial compliance will be limited (the file format and compression ratio may vary from vendor to vendor), but in order to avoid the current limitations of DICOM in pathology, a universal viewer, accepting most available file formats existing today, will be selected for the neuropathology platform. 

Recently, other technologic improvements have been carried out to reduce timing consumed in telepathology. 

Firstly, currently scanners are becoming very fast. Now it is possible to perform whole slide scans in less than 1 minute [5] and, consequently, it is possible to scan numerous slides through digital scanners in an acceptable time. 

Scanning time and quality are issues that need to be addressed and handled with care. Following College of American Pathologists’ (CAP) recommendations, the neuropathology network will begin with a validation study of 60 cases, plus 20 cases for each different scanner participating in the network, in order to confirm the diagnosis performance. 

Secondly, the time invested in sending images is no longer a problem [10]. WSI allows the transmission of data to neuropathologists around the world within a few minutes after a slide is created. This technology allows rapid second opinions and consultations on challenging diagnostic cases. 

Thirdly, it is possible to share images with a large number of colleagues at the same time [9]. By using computer conferencing tools, images can be viewed by multiple parties simultaneously, similarly to what happens with a multiheaded microscope. Therefore, a WSI on a single server is accessed by multiple users who can dynamically pass control over the slides among themselves and can see digital markings and annotations added to it. 

Fourthly, it seems possible to go further in the diagnostic field. In fact, it has been demonstrated that these technologies are suitable for performing pathological diagnoses [6]. The WSI system has been validated for primary diagnosis in surgical pathology [11] and, in that regard, the American College of Pathology has elaborated a practical guide for validating WSI systems for diagnostic work. The WSI for primary diagnosis has received approval by the European Union as well [12]. In fact, the United States is so far the only industrialized Western country to not have approved whole-slide imaging for primary diagnosis. 

Fifthly, the storage amount needed in a digital neuropathology system server to allocate the daily work exceeds the capacity of the current servers in most hospitals. 

## Why establish a neuropathology network in the European context? 

Europe is the expected geographic context for communication and relationship among European neuropathologists, and clinical consultancy should be reinforced as much as possible in this context. Considered as a whole, neuropathology is a very suitable discipline for the launching of convergent cross-border European initiatives related to diagnosis, research, and teaching. 

There are already relevant European initiatives in the field of telepathology networking. For example, *Euro-telepath* has the goal of developing standards for digital pathology [13, 14, 15] and Academia and Industry Collaboration for Digital Pathology (AIDPATH), which was created in 2013, has the objective of exploiting emerging digital pathology technologies, including in universities and industry. 

In addition, a European diagnostic nephropathology network has been established [16, 17]. According to its developers, the use of WSI brings advantages such as efficiency, facilitation of pathology review in a clinical trial setting, improved intraobserver and interobserver reproducibility, and web-based consensus meetings. 

Neuropathology networking should evolve to create a large-scale consortium of interconnected facilities from different hospitals and institutions across Europe: some of them would mostly send cases for consultancy whereas some others would mostly receive cases for diagnosis. With the progressive availability of scanners, it is likely that the development of this transnational network will soon become a reality. 

## Points of discussion in relation to a transnational consultancy network in neuropathology. Its potential host: who and where? 

Several limitations should be considered in the implementation of a digital neuropathology consultancy network. 

Firstly, which institutions should start and support this initiative? Certainly, it should be a European organ concerned with neuropathology and endowed with appropriate personnel and financial resources. In that regard, it is possible that different financing options may arise, as for example company sponsorship and research project funding. Financial support coming from Europe would be welcomed. 

Secondly, is this initiative financially sustainable? The needs of personnel and infrastructure should be kept to a minimum. It should be clear that we are not proposing the implementation of hospital digital neuropathology systems, but the EURO-CNS organization only need to connect neuropathologists or institutions already using digital systems. Incidentally, it has been estimated that the implementation of digital pathology could result in cost savings [4]. However, estimations about how much financial support would be needed are essential and should be considered before launching this digital neuropathology initiative. 

Certainly, operational costs for digital pathology are high. This project may benefit, at least initially, from the efforts already made by many pathology departments to implement digital pathology scanning solutions. This means that in order to create the European Network of Neuropathology, no initial investment in scanners would be needed. Digital slide storage costs will only become significant if thousands of slides are exchanged using this network, and this will mean that the project has already succeeded, and in this case, different financing options may arise (e.g., company sponsorship, case fee, research project funding). 

Technical support is also a very important aspect that must be considered. Several easy-to-use digital slide platforms exists, and in case limited funds are available, a digital network could start with a low cost license (3,000 Euro/year) of an internet-based digital slide solutions, that allows digital slide format from most vendors. This annual fee will include all needed technical support. 

In the first phase of the project, some other operational costs may be even higher than those directly related to digital slides, like user support or case editing and formatting. These costs can also be significantly reduced if the adequate platform, including chat or remote control options, is selected. 

On the other hand, how should consultants be compensated for their work and the time they will spend solving the diagnostic problems of their colleagues? It seems reasonable that the honor of being a consultant should be complemented with some kind of financial reward. It is likely that, once the system becomes active, there will be an increasing demand. The latter, eventually, might translate into a source of income for the EURO-CNS. Certainly, a large-scale pan-European implementation of digital neuropathology does not have to be an expensive question of creating an attractive system and fomenting interest in the initiative among all those involved. 

Thirdly, legal, privacy, security, and confidentiality issues must be considered and solved before launching the network [13]. 

There is no specific European Union (EU) legislation on telemedicine. In Europe, Member States are primarily responsible for the organization, financing, and delivery of healthcare. Currently, does not exist an EU legislation specifically on telemedicine. Telepathology as an aspect of telemedicine falls within the scope of Directive 2011/24/EU on the application of patients’ rights in cross-border healthcare. Moreover, the European Commission (EC) Commission Staff Working Document (06.12.2012) was written to develop the existing EU legal framework applicable to telemedicine. In order to provide telemedicine cross-border within the EU, healthcare professionals have to look for the responses to the licensing, data protection, reimbursement, and liability. 

According to several directives of the EU, in most Member States, the competence to accredit professionals wishing to deliver health services is delegated to an appointed licensing or registration body. The telemedicine provider should comply with the authorization and registration requirements of his or her Member State. If the healthcare professional complies with the legislation applicable to the taking up and provision of an information society service in his or her Member State of establishment, he will in principle be free to provide these services in other Member States. Upon being licensed/registered, the health professional will have to abide by the rules and regulations established by the licensing authority and be subject to disciplinary sanctions in case of nonobservance. 

Health data are very sensitive. The EU adopted the e-Privacy Directive 2002/58/EC, which was aimed at ensuring the protection of personal data in the field of telecommunications. The EU Data Protection Directive 1995/46/EC is applicable to the automated processing of personal data. Obviously, it is need the professional secrecy and explicit consent from the patient. It is an obligation of the data controller to implement appropriate security measures to protect personal data. The data controller should disclose the purposes for which the data are intended. The data controller is also obliged to implement adequate technical and organizational measures against unlawful access, accidental loss, destruction and alteration of data. 

With respect to reimbursement, it is up to the Member States to decide whether telemedicine must be reimbursed. According to the mentioned EC Commission Staff Working Document, telemedicine services or patients receiving healthcare in another Member State have to be reimbursed. 

No EU legislation about medical liability exists. Medical liability is regulated at the Member State level, and the complexity and diversity of liability rules in the Member States regarding the provision of healthcare are considerable. 

Fourthly, when paraffin blocks are not available, the consultant inform know the case sender as to which new studies should be performed on the paraffin bocks, and then the new scanned slides may be sent to the consultant. Alternatively, the consultant could receive the paraffin blocks to further study the case. 

Finally, we think that all these shortcuts can be solved and the network can be successfully developed. To that end, a preliminary study by a group of institutions with WSI scanners has already been started. We have a historical opportunity to create a European network in neuropathology. Thanks to digital technology, neuropathologists have to confront challenging clinical cases in isolation. On the contrary, they may be interconnected by a European neuropathology organization *with a true interest in this matter. Inasmuch as we are convinced of the advantages of the digital revolution in medicine, a reliable European digital neuropathology network should be created for the patients’ benefit.*

## Acknowledgment 

We thank Dr. Aurelio Ariza, Department of Pathology, Hospital Germans Trias i Pujol, Autonomous University of Barcelona, Badalona, Barcelona, for his helpful suggestions. 

## Conflict of interest 

The authors declare that they have no conflicts of interest. 
